# Supplementary material for: A Novel P53 Nanomedicine Reduces Immunosuppression and Augments Anti-PD-1 Therapy for Non-Small Cell Lung Cancer in Syngeneic Mouse Models
Source: Cells. 2022 Oct 31;11(21):3434. doi: 10.3390/cells11213434 (PMC9654894; doi:10.3390/cells11213434)
Supplement: Supplementary file 1 [file cells-11-03434-s001.zip › cells-1924087-supplementary.pdf]

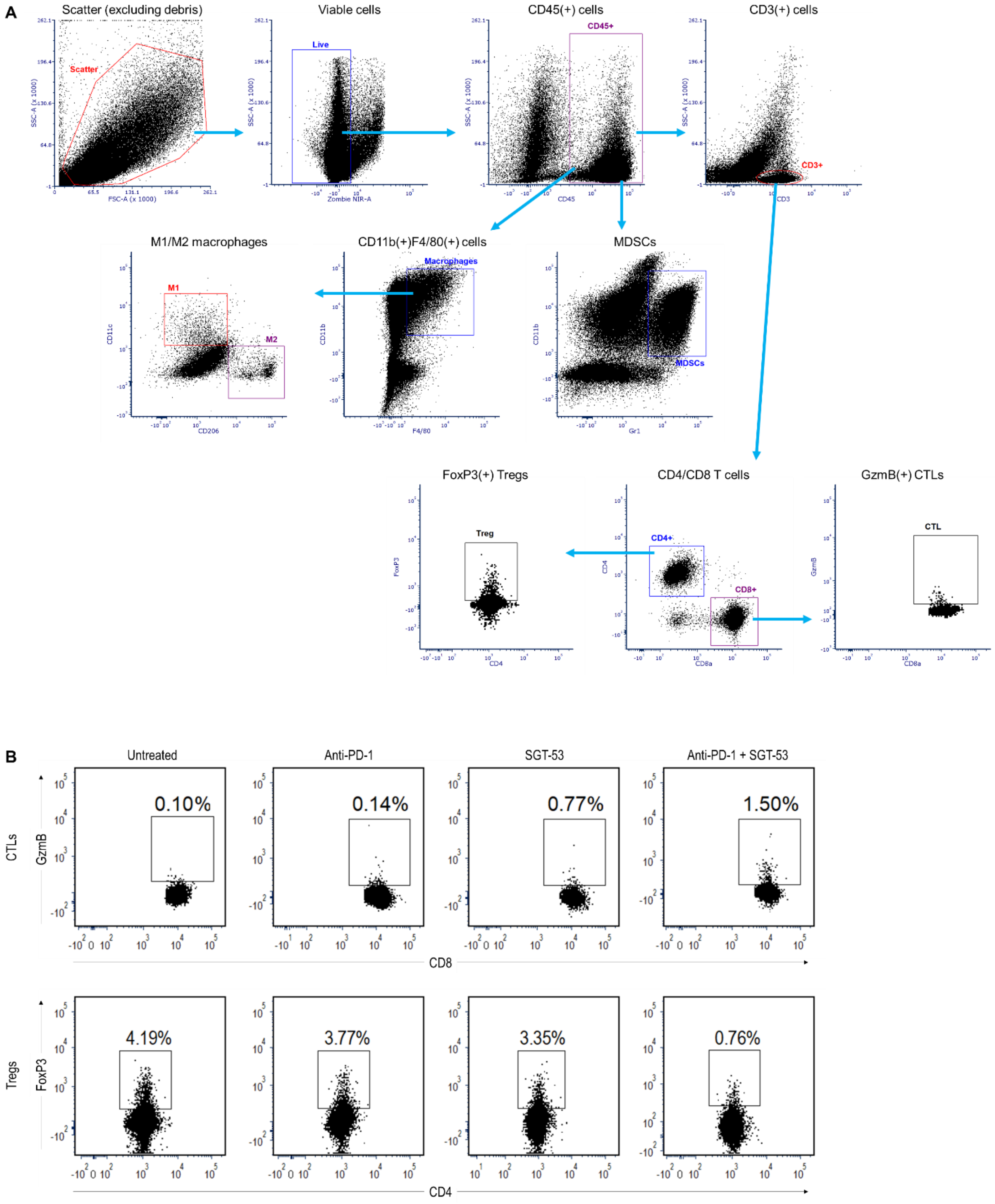
A
Scatter (excluding debris)
Scatter
Viable cells
Live
CD45(+) cells
CD45+
CD3(+) cells
CD3+
M1/M2 macrophages
M1
M2
CD11b(+)F4/80(+) cells
Macrophages
MDSCs
MDSCs
FoxP3(+) Tregs
Treg
CD4/CD8 T cells
CD4+
CD8+
GzmB(+) CTLs
CTL
B
Untreated
Anti-PD-1
SGT-53
Anti-PD-1 + SGT-53
CTLs
GzmB
0.10%
0.14%
0.77%
1.50%
CD8
Tregs
FoxP3
4.19%
3.77%
3.35%
0.76%
CD4

C

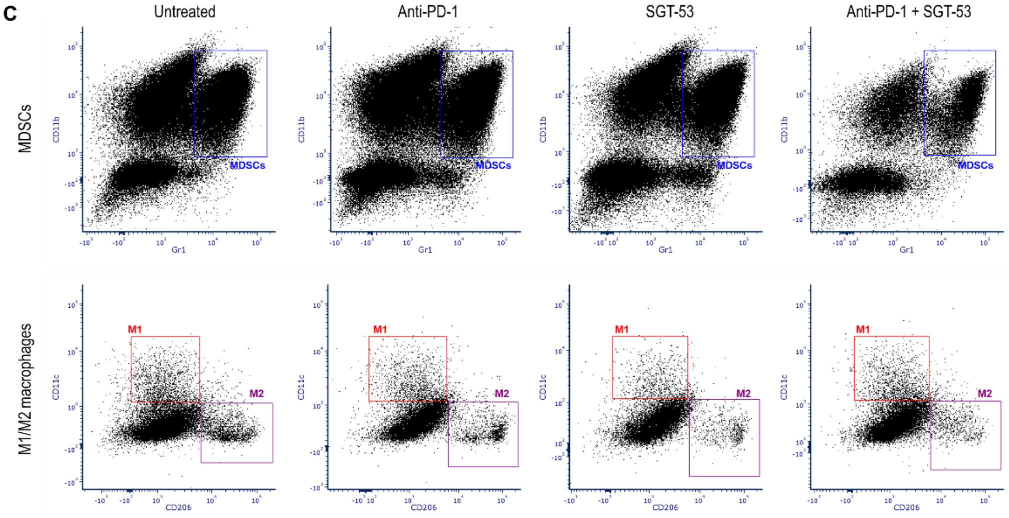

Figure S1. Flow representation with full gate for Figure 4. (**A**) Flow cytometry gating strategy used to define CTLs (CD45$^+$CD3$^+$CD8$^+$GzmB$^+$), Tregs (CD45$^+$CD3$^+$CD4$^+$FoxP3$^+$), MDSCs (CD45$^+$CD11b$^+$Gr1$^+$), M1 macrophages (CD45$^+$CD11b$^+$F4/80$^+$CD11c$^+$), and M2 macrophages (CD45$^+$CD11b$^+$F4/80$^+$CD206$^+$) among live (Zombie-NIR$^-$) tumor cells for Figure 4C,4D,4E,4G, and 4H. Representative example for (**B**) Figure 4C and 4D and (**C**) Figure 4E,4G, and 4H.

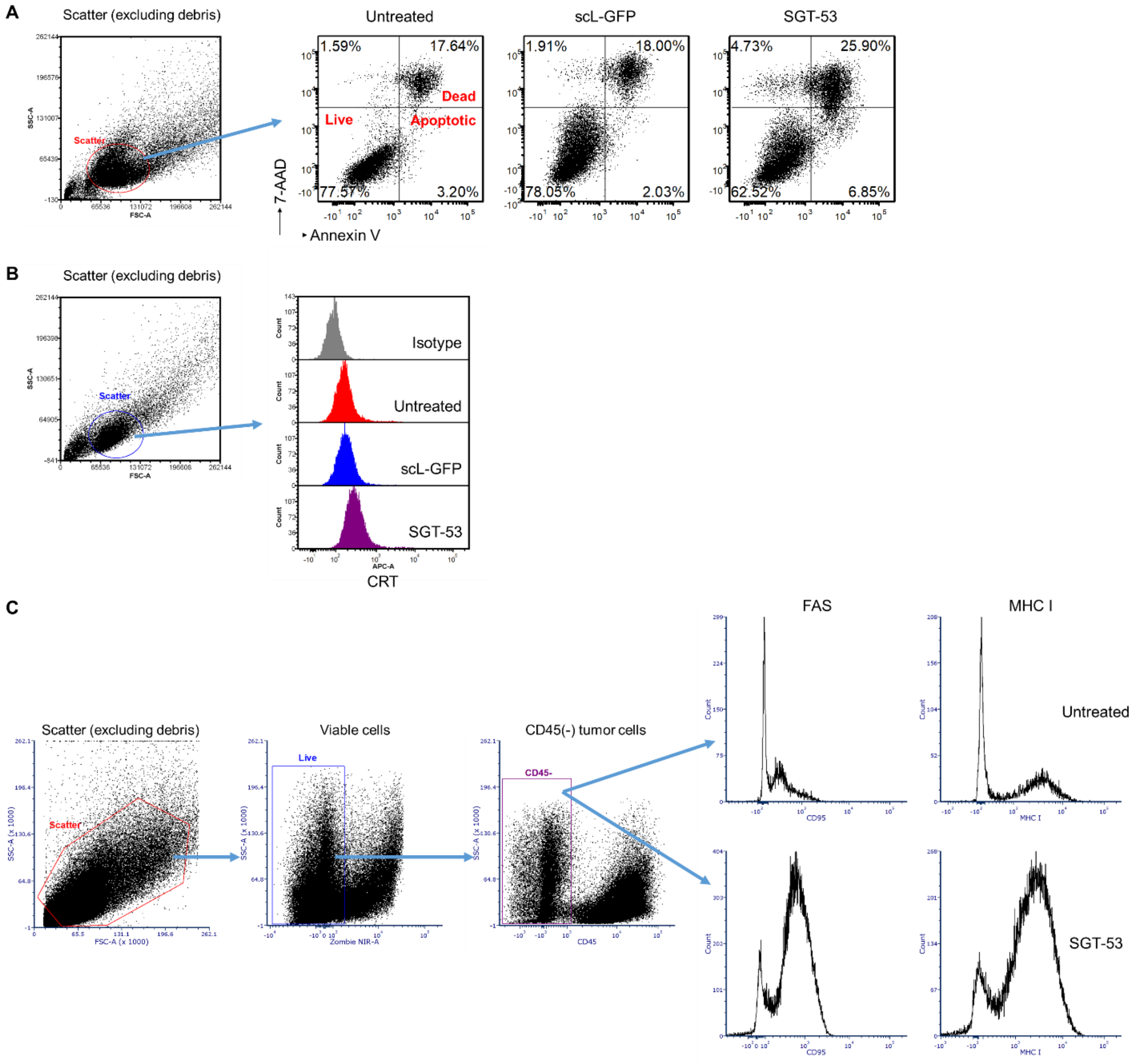

Figure S2. Flow representation with full gate for Figure 5. Flow cytometry gating strategy and representative example for (**A**) Figure 5A and (**B**) Figure 5B. (**C**) Flow cytometry gating strategy used to define tumor cells (Zombie-NIR^{-}CD45^{-}) and representative example for Figure 5E.

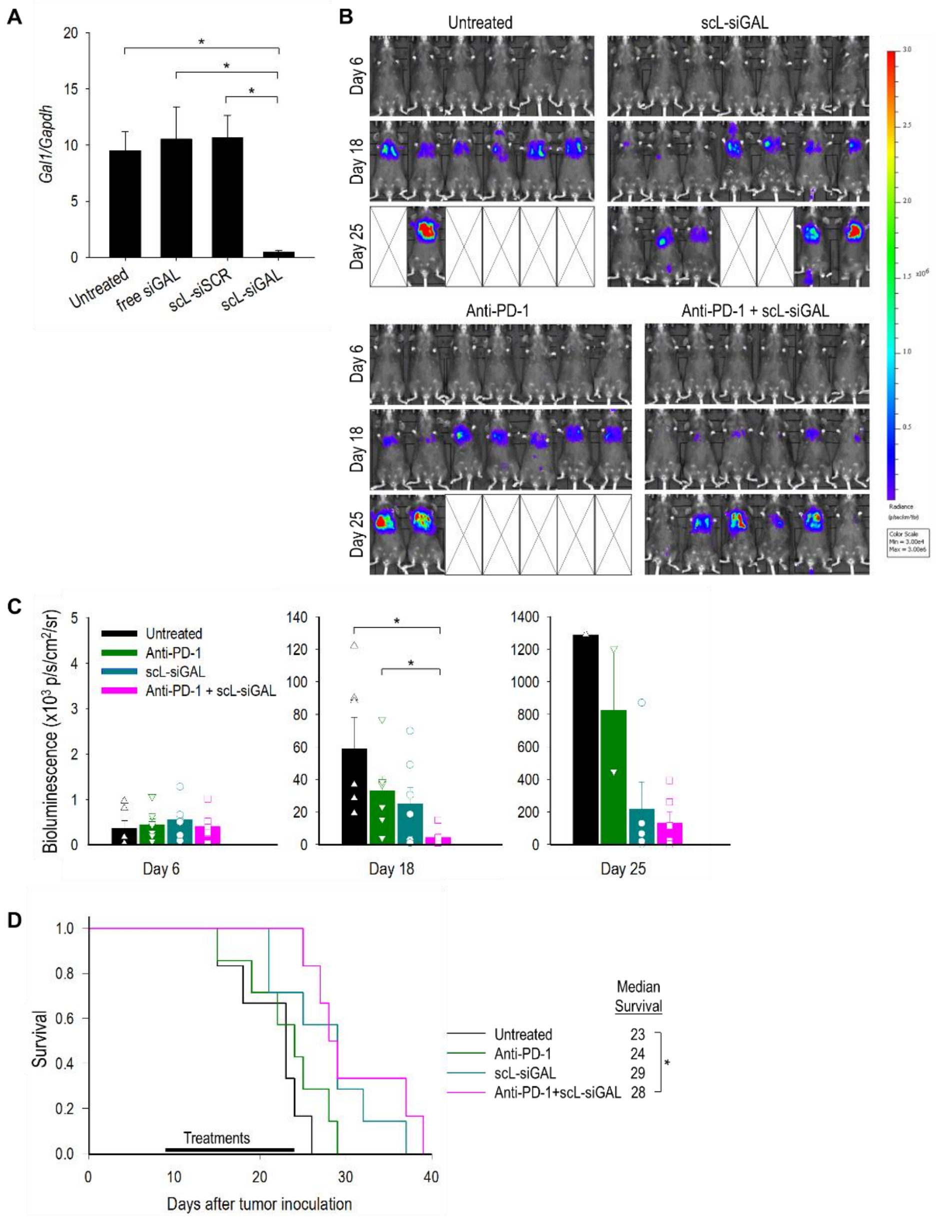

Figure S3. siRNA-mediated knockdown of Gal-1 enhances the efficacy of anti-PD-1 therapy in a metastatic LL/2-luc tumor model. (**A**) LL/2 cells were transfected with either siRNA targeting Gal-1 without the delivery system (free siGAL), scL encapsulating scrambled control siRNA (scL-siSCR), or scL encapsulating Gal-1 siRNA (scL-siGAL) at 50 nM siRNA concentration. Twenty-four hours after transfection, Gal-1 mRNA level was measured by quantitative RT-PCR in triplicate. *p<0.05. For *in vivo* study, C57BL/6 mice with metastatic LL/2-luc tumors were randomized to therapy with anti-PD-1 (200 µg, IP) only, scL-siGAL (30 µg siRNA, IV) only, or the combination of both agents. n = 6-7/group. Untreated control mice or mice treated with anti-PD-1 only are the same as in Figure 2. (**B**) Bioluminescence images of metastatic lung cancer are shown. Bioluminescence signals, shown in a color map, correlate with tumor sizes. Red color: stronger signal, violet color: weaker signal. (**C**) Bioluminescence intensities of tumors were plotted. *p<0.05. (**D**) Kaplan–Meier survival curves of mice. Log-Rank test, *p<0.05.
